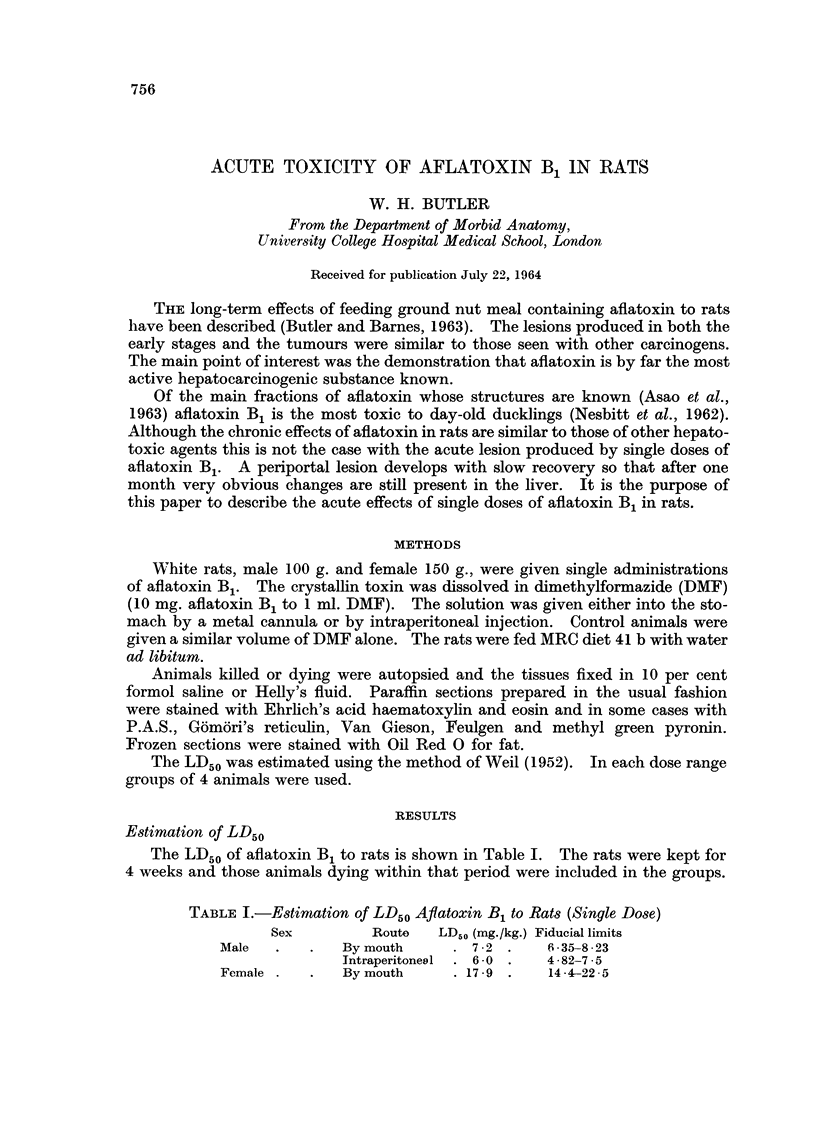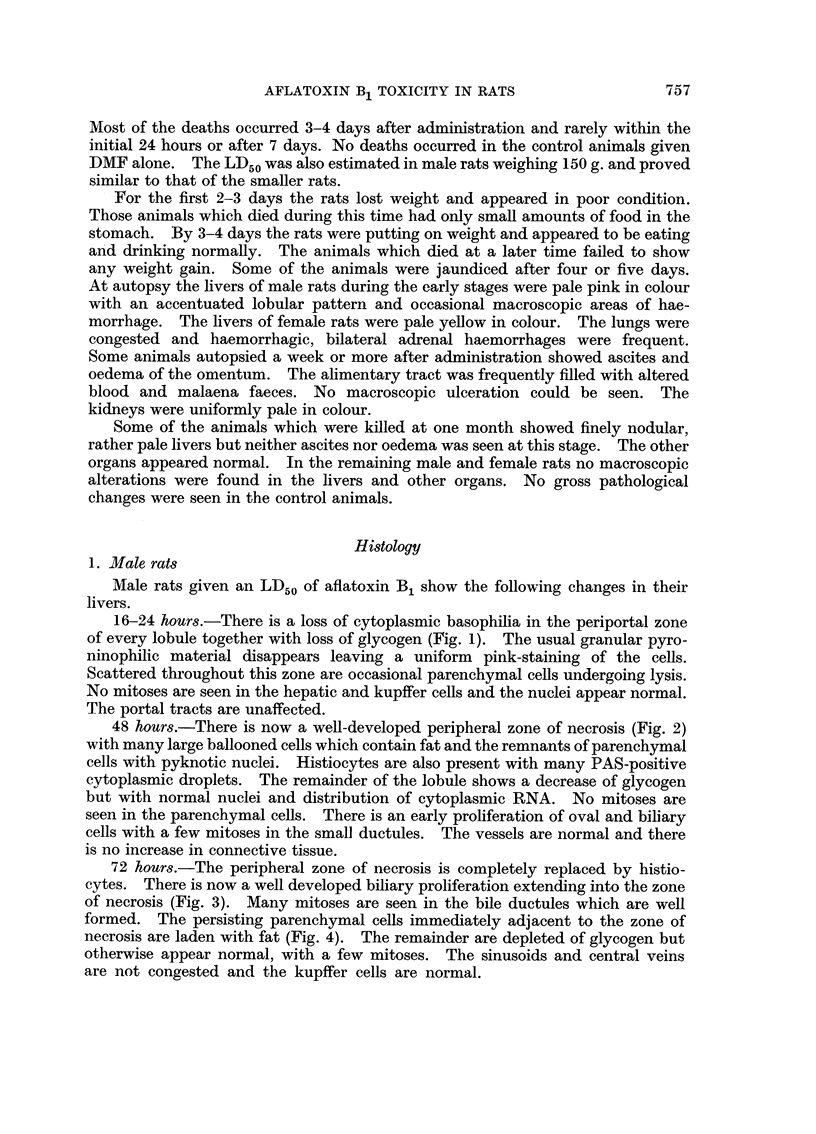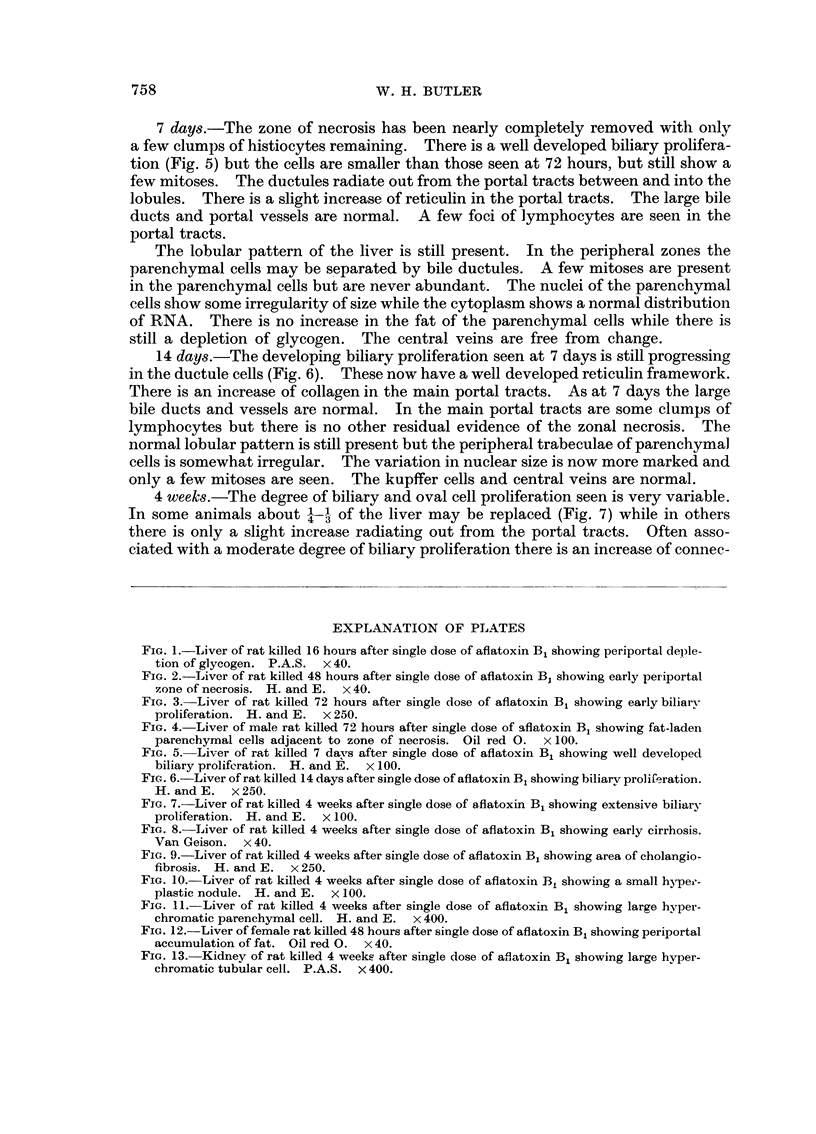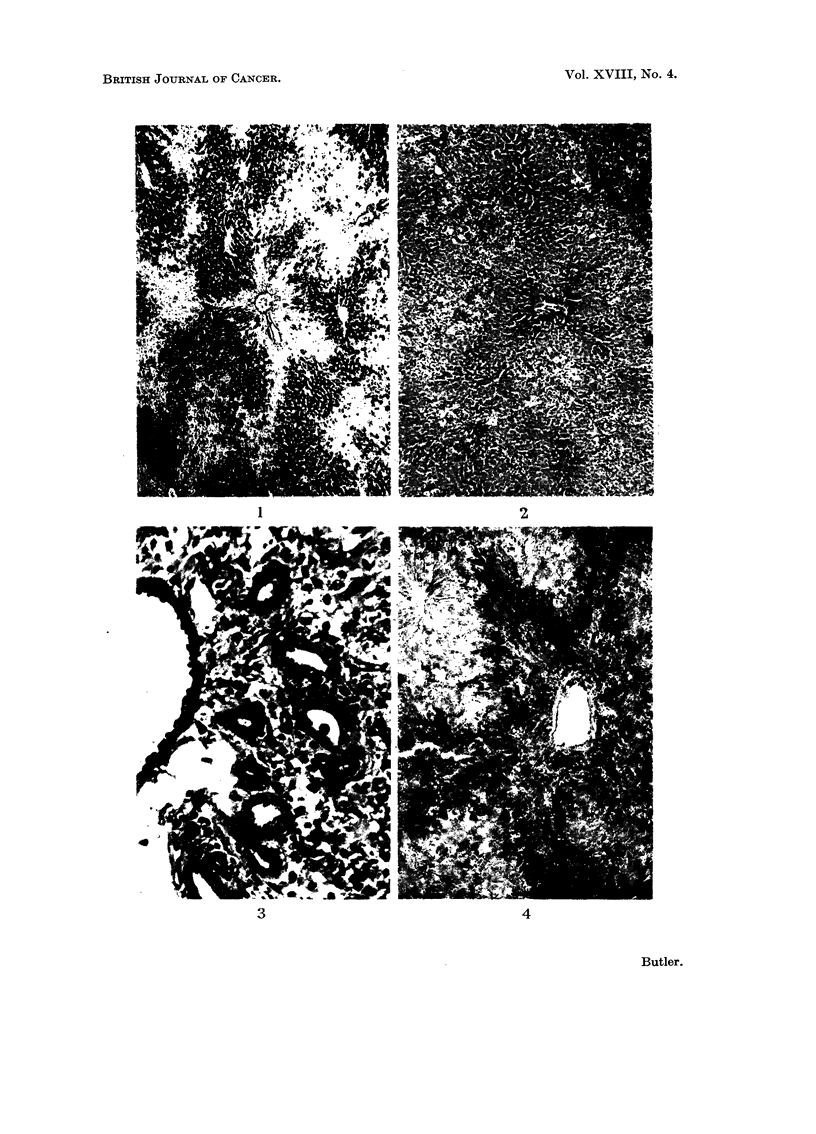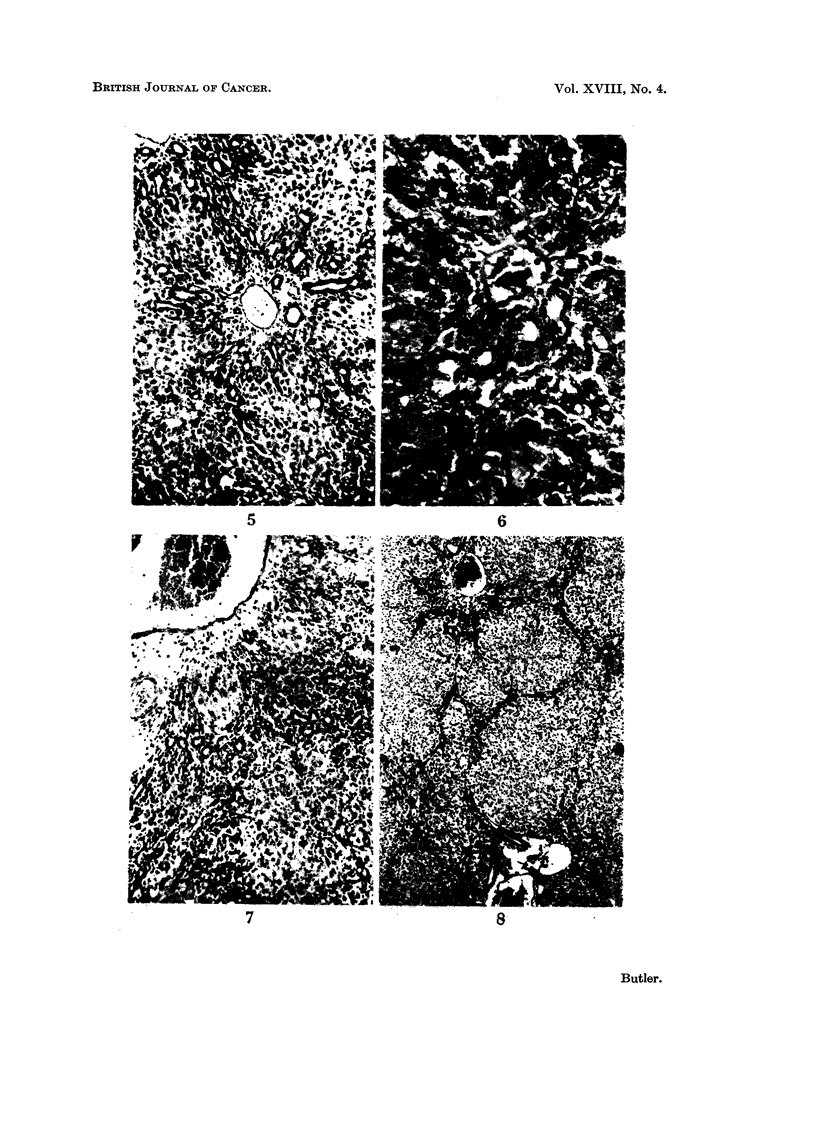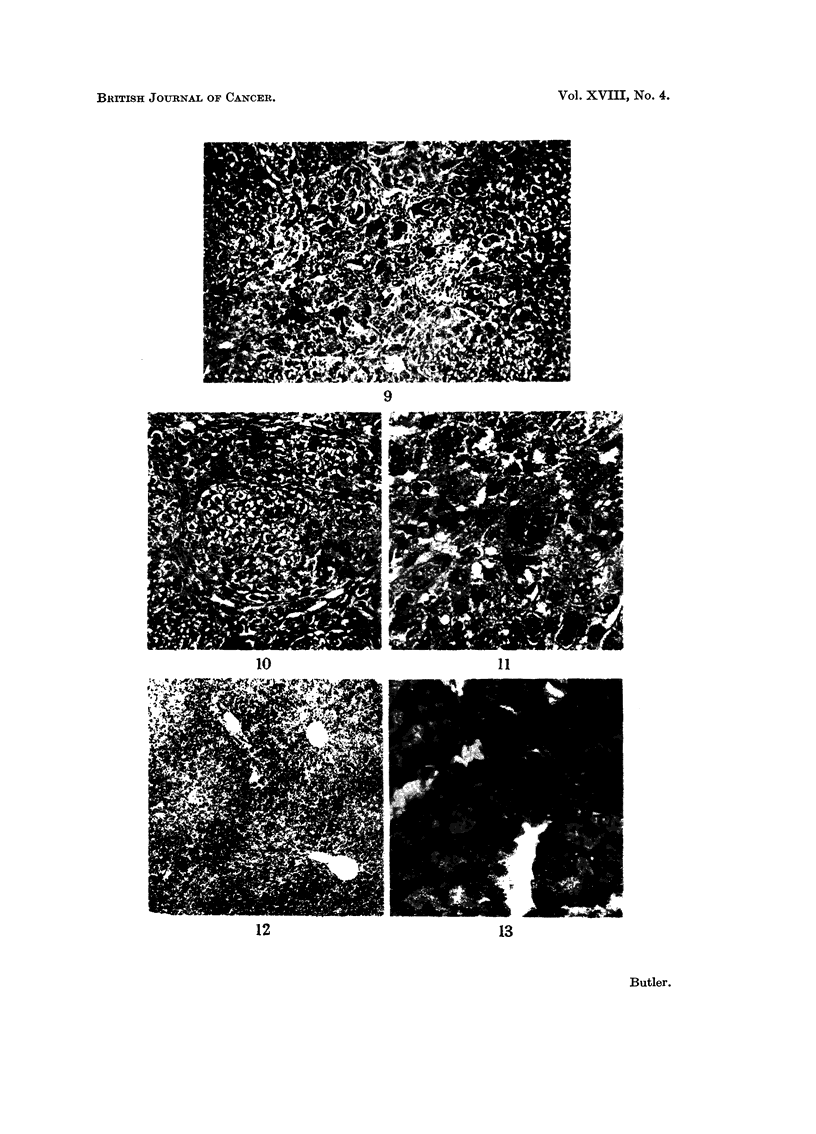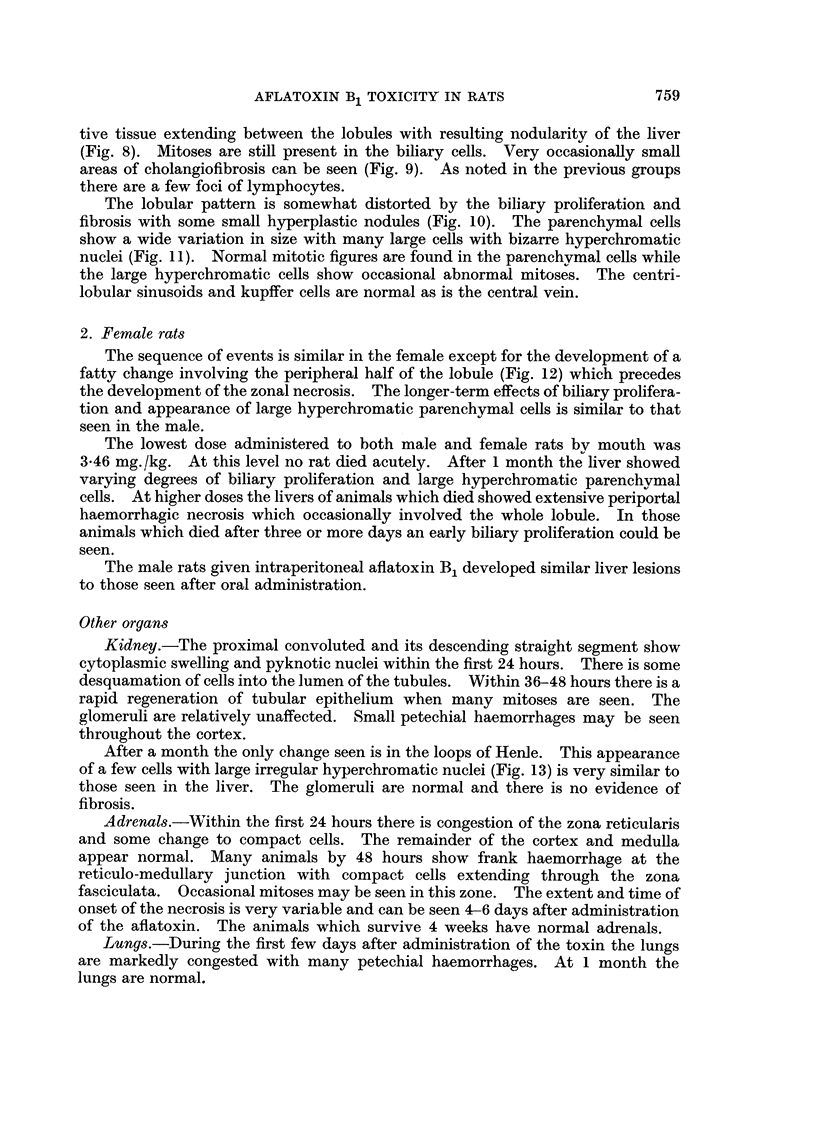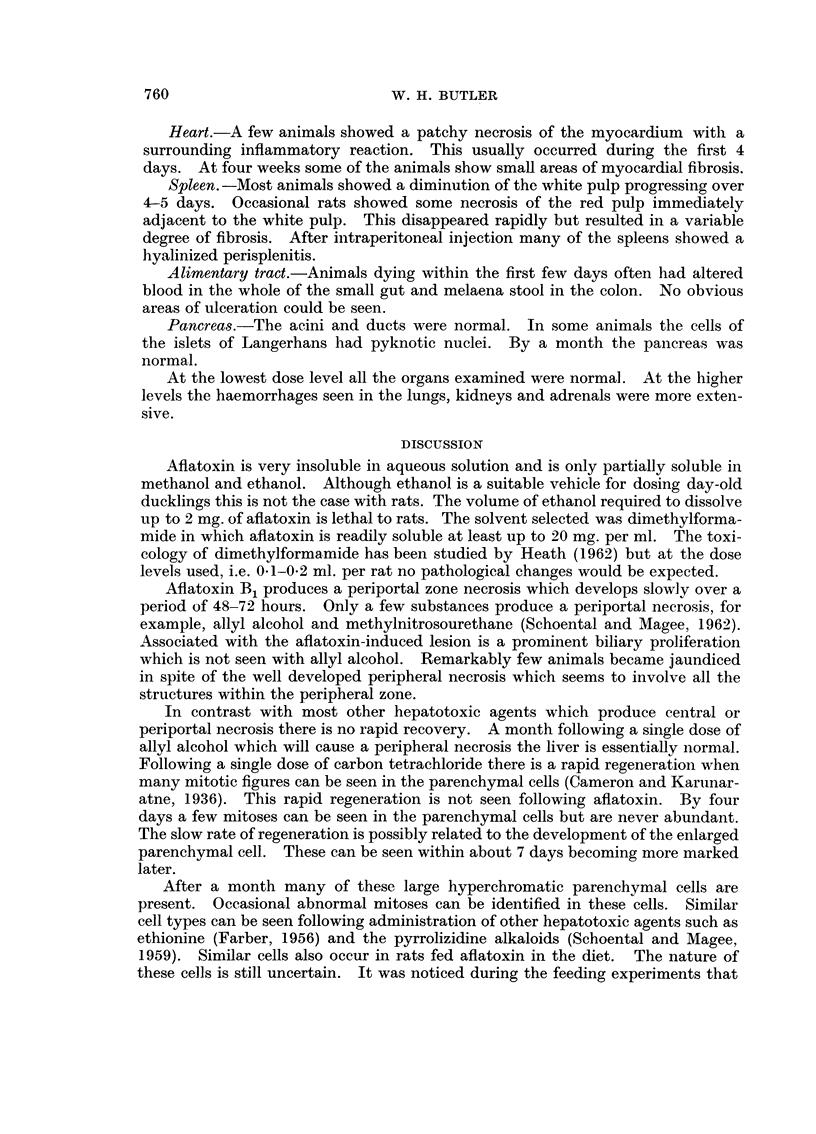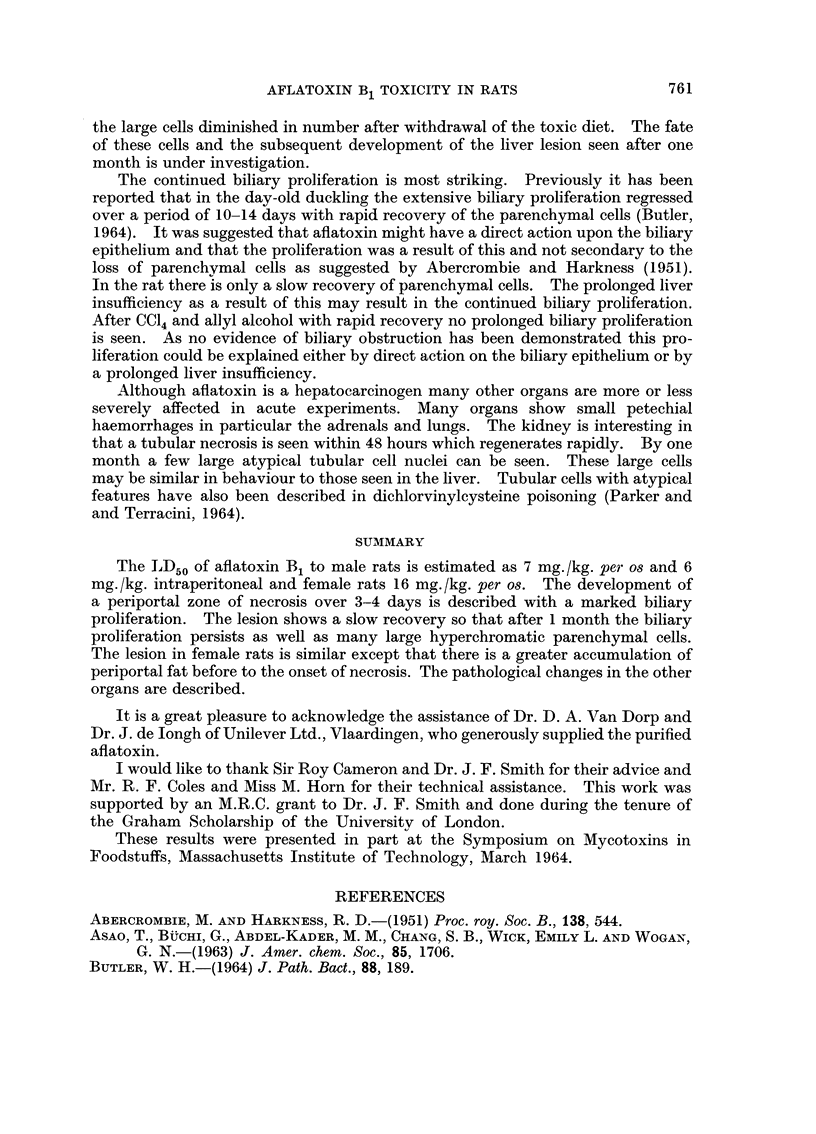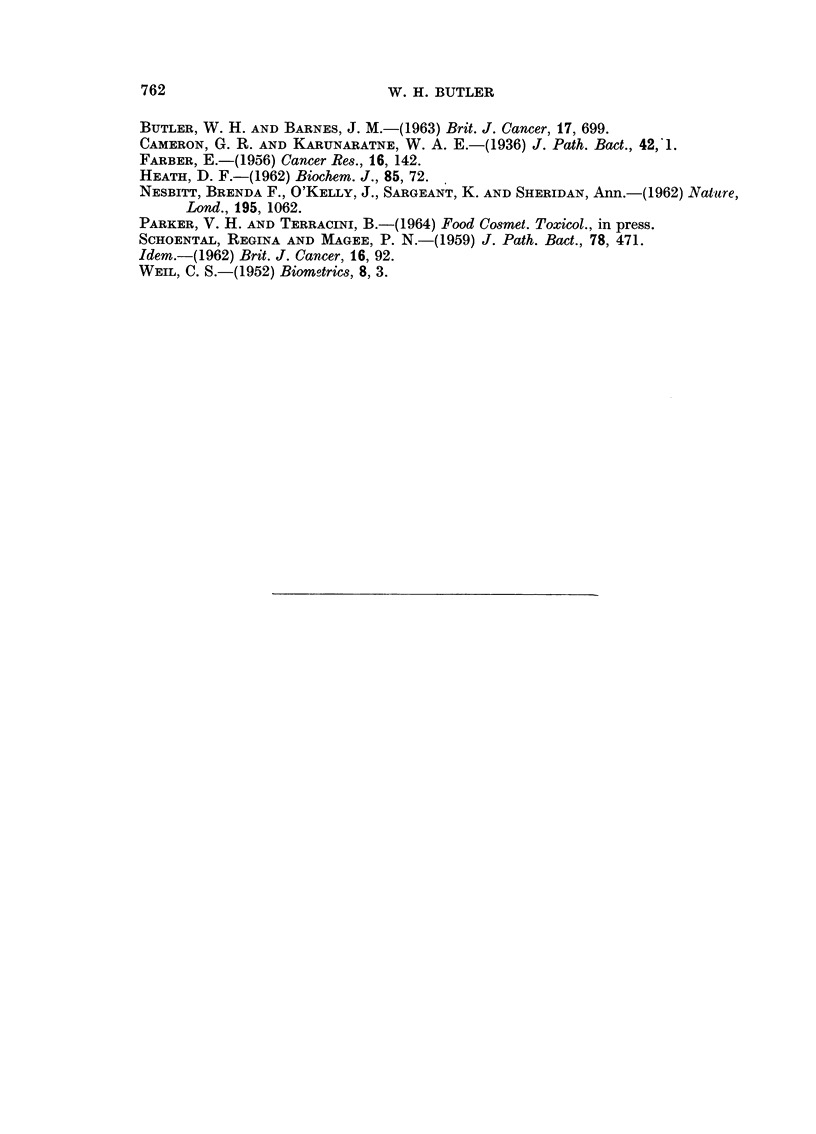# Acute Toxicity of Aflatoxin B_1_ in Rats

**DOI:** 10.1038/bjc.1964.87

**Published:** 1964-12

**Authors:** W. H. Butler

## Abstract

**Images:**


### ACUTE TOXICITY OF AFLATOXIN $B_1$ IN RATS

W. H. BUTLER
*From the Department of Morbid Anatomy, University College Hospital Medical School, London*

Received for publication July 22, 1964

THE long-term effects of feeding ground nut meal containing aflatoxin to rats have been described (Butler and Barnes, 1963). The lesions produced in both the early stages and the tumours were similar to those seen with other carcinogens. The main point of interest was the demonstration that aflatoxin is by far the most active hepatocarcinogenic substance known.

Of the main fractions of aflatoxin whose structures are known (Asao *et al.*, 1963) aflatoxin $B_1$ is the most toxic to day-old ducklings (Nesbitt *et al.*, 1962). Although the chronic effects of aflatoxin in rats are similar to those of other hepatotoxic agents this is not the case with the acute lesion produced by single doses of aflatoxin $B_1$. A periportal lesion develops with slow recovery so that after one month very obvious changes are still present in the liver. It is the purpose of this paper to describe the acute effects of single doses of aflatoxin $B_1$ in rats.

### METHODS

White rats, male 100 g. and female 150 g., were given single administrations of aflatoxin $B_1$. The crystallin toxin was dissolved in dimethylformazide (DMF) (10 mg. aflatoxin $B_1$ to 1 ml. DMF). The solution was given either into the stomach by a metal cannula or by intraperitoneal injection. Control animals were given a similar volume of DMF alone. The rats were fed MRC diet 41 b with water *ad libitum.*

Animals killed or dying were autopsied and the tissues fixed in 10 per cent formol saline or Helly's fluid. Paraffin sections prepared in the usual fashion were stained with Ehrlich's acid haematoxylin and eosin and in some cases with P.A.S., Gömöri's reticulin, Van Gieson, Feulgen and methyl green pyronin. Frozen sections were stained with Oil Red O for fat.

The $LD_{50}$ was estimated using the method of Weil (1952). In each dose range groups of 4 animals were used.

### RESULTS

### *Estimation of $LD_{50}$*

The $LD_{50}$ of aflatoxin $B_1$ to rats is shown in Table I. The rats were kept for 4 weeks and those animals dying within that period were included in the groups.

TABLE I.—*Estimation of $LD_{50}$ Aflatoxin $B_1$ to Rats (Single Dose)*

| Sex | Route | $LD_{50}$ (mg./kg.) | Fiducial limits |
|---|---|---|---|
| Male | By mouth | 7·2 | 6·35–8·23 |
| | Intraperitoneal | 6·0 | 4·82–7·5 |
| Female | By mouth | 17·9 | 14·4–22·5 |

Most of the deaths occurred 3–4 days after administration and rarely within the initial 24 hours or after 7 days. No deaths occurred in the control animals given DMF alone. The $LD_{50}$ was also estimated in male rats weighing 150 g. and proved similar to that of the smaller rats.

For the first 2–3 days the rats lost weight and appeared in poor condition. Those animals which died during this time had only small amounts of food in the stomach. By 3–4 days the rats were putting on weight and appeared to be eating and drinking normally. The animals which died at a later time failed to show any weight gain. Some of the animals were jaundiced after four or five days. At autopsy the livers of male rats during the early stages were pale pink in colour with an accentuated lobular pattern and occasional macroscopic areas of haemorrhage. The livers of female rats were pale yellow in colour. The lungs were congested and haemorrhagic, bilateral adrenal haemorrhages were frequent. Some animals autopsied a week or more after administration showed ascites and oedema of the omentum. The alimentary tract was frequently filled with altered blood and malaena faeces. No macroscopic ulceration could be seen. The kidneys were uniformly pale in colour.

Some of the animals which were killed at one month showed finely nodular, rather pale livers but neither ascites nor oedema was seen at this stage. The other organs appeared normal. In the remaining male and female rats no macroscopic alterations were found in the livers and other organs. No gross pathological changes were seen in the control animals.

### *Histology*

1. *Male rats*

Male rats given an $LD_{50}$ of aflatoxin $B_1$ show the following changes in their livers.

16–24 *hours.*—There is a loss of cytoplasmic basophilia in the periportal zone of every lobule together with loss of glycogen (Fig. 1). The usual granular pyroninophilic material disappears leaving a uniform pink-staining of the cells. Scattered throughout this zone are occasional parenchymal cells undergoing lysis. No mitoses are seen in the hepatic and kupffer cells and the nuclei appear normal. The portal tracts are unaffected.

48 *hours.*—There is now a well-developed peripheral zone of necrosis (Fig. 2) with many large ballooned cells which contain fat and the remnants of parenchymal cells with pyknotic nuclei. Histiocytes are also present with many PAS-positive cytoplasmic droplets. The remainder of the lobule shows a decrease of glycogen but with normal nuclei and distribution of cytoplasmic RNA. No mitoses are seen in the parenchymal cells. There is an early proliferation of oval and biliary cells with a few mitoses in the small ductules. The vessels are normal and there is no increase in connective tissue.

72 *hours.*—The peripheral zone of necrosis is completely replaced by histiocytes. There is now a well developed biliary proliferation extending into the zone of necrosis (Fig. 3). Many mitoses are seen in the bile ductules which are well formed. The persisting parenchymal cells immediately adjacent to the zone of necrosis are laden with fat (Fig. 4). The remainder are depleted of glycogen but otherwise appear normal, with a few mitoses. The sinusoids and central veins are not congested and the kupffer cells are normal.

7 *days*.—The zone of necrosis has been nearly completely removed with only a few clumps of histiocytes remaining. There is a well developed biliary proliferation (Fig. 5) but the cells are smaller than those seen at 72 hours, but still show a few mitoses. The ductules radiate out from the portal tracts between and into the lobules. There is a slight increase of reticulin in the portal tracts. The large bile ducts and portal vessels are normal. A few foci of lymphocytes are seen in the portal tracts.

The lobular pattern of the liver is still present. In the peripheral zones the parenchymal cells may be separated by bile ductules. A few mitoses are present in the parenchymal cells but are never abundant. The nuclei of the parenchymal cells show some irregularity of size while the cytoplasm shows a normal distribution of RNA. There is no increase in the fat of the parenchymal cells while there is still a depletion of glycogen. The central veins are free from change.

14 *days*.—The developing biliary proliferation seen at 7 days is still progressing in the ductule cells (Fig. 6). These now have a well developed reticulin framework. There is an increase of collagen in the main portal tracts. As at 7 days the large bile ducts and vessels are normal. In the main portal tracts are some clumps of lymphocytes but there is no other residual evidence of the zonal necrosis. The normal lobular pattern is still present but the peripheral trabeculae of parenchymal cells is somewhat irregular. The variation in nuclear size is now more marked and only a few mitoses are seen. The kupffer cells and central veins are normal.

4 *weeks*.—The degree of biliary and oval cell proliferation seen is very variable. In some animals about $\frac{1}{4}$–$\frac{1}{3}$ of the liver may be replaced (Fig. 7) while in others there is only a slight increase radiating out from the portal tracts. Often associated with a moderate degree of biliary proliferation there is an increase of connec-

---

EXPLANATION OF PLATES

FIG. 1.—Liver of rat killed 16 hours after single dose of aflatoxin $B_1$ showing periportal depletion of glycogen. P.A.S. ×40.

FIG. 2.—Liver of rat killed 48 hours after single dose of aflatoxin $B_1$ showing early periportal zone of necrosis. H. and E. ×40.

FIG. 3.—Liver of rat killed 72 hours after single dose of aflatoxin $B_1$ showing early biliary proliferation. H. and E. ×250.

FIG. 4.—Liver of male rat killed 72 hours after single dose of aflatoxin $B_1$ showing fat-laden parenchymal cells adjacent to zone of necrosis. Oil red O. ×100.

FIG. 5.—Liver of rat killed 7 days after single dose of aflatoxin $B_1$ showing well developed biliary proliferation. H. and E. ×100.

FIG. 6.—Liver of rat killed 14 days after single dose of aflatoxin $B_1$ showing biliary proliferation. H. and E. ×250.

FIG. 7.—Liver of rat killed 4 weeks after single dose of aflatoxin $B_1$ showing extensive biliary proliferation. H. and E. ×100.

FIG. 8.—Liver of rat killed 4 weeks after single dose of aflatoxin $B_1$ showing early cirrhosis. Van Geison. ×40.

FIG. 9.—Liver of rat killed 4 weeks after single dose of aflatoxin $B_1$ showing area of cholangiofibrosis. H. and E. ×250.

FIG. 10.—Liver of rat killed 4 weeks after single dose of aflatoxin $B_1$ showing a small hyperplastic nodule. H. and E. ×100.

FIG. 11.—Liver of rat killed 4 weeks after single dose of aflatoxin $B_1$ showing large hyperchromatic parenchymal cell. H. and E. ×400.

FIG. 12.—Liver of female rat killed 48 hours after single dose of aflatoxin $B_1$ showing periportal accumulation of fat. Oil red O. ×40.

FIG. 13.—Kidney of rat killed 4 weeks after single dose of aflatoxin $B_1$ showing large hyperchromatic tubular cell. P.A.S. ×400.

1

2

3

4

Butler.

5

6

7

8

Butler.

9

10

11

12

13

Butler.

tive tissue extending between the lobules with resulting nodularity of the liver (Fig. 8). Mitoses are still present in the biliary cells. Very occasionally small areas of cholangiofibrosis can be seen (Fig. 9). As noted in the previous groups there are a few foci of lymphocytes.

The lobular pattern is somewhat distorted by the biliary proliferation and fibrosis with some small hyperplastic nodules (Fig. 10). The parenchymal cells show a wide variation in size with many large cells with bizarre hyperchromatic nuclei (Fig. 11). Normal mitotic figures are found in the parenchymal cells while the large hyperchromatic cells show occasional abnormal mitoses. The centrilobular sinusoids and kupffer cells are normal as is the central vein.

2. *Female rats*

The sequence of events is similar in the female except for the development of a fatty change involving the peripheral half of the lobule (Fig. 12) which precedes the development of the zonal necrosis. The longer-term effects of biliary proliferation and appearance of large hyperchromatic parenchymal cells is similar to that seen in the male.

The lowest dose administered to both male and female rats by mouth was 3·46 mg./kg. At this level no rat died acutely. After 1 month the liver showed varying degrees of biliary proliferation and large hyperchromatic parenchymal cells. At higher doses the livers of animals which died showed extensive periportal haemorrhagic necrosis which occasionally involved the whole lobule. In those animals which died after three or more days an early biliary proliferation could be seen.

The male rats given intraperitoneal aflatoxin $B_1$ developed similar liver lesions to those seen after oral administration.

*Other organs*

*Kidney*.—The proximal convoluted and its descending straight segment show cytoplasmic swelling and pyknotic nuclei within the first 24 hours. There is some desquamation of cells into the lumen of the tubules. Within 36–48 hours there is a rapid regeneration of tubular epithelium when many mitoses are seen. The glomeruli are relatively unaffected. Small petechial haemorrhages may be seen throughout the cortex.

After a month the only change seen is in the loops of Henle. This appearance of a few cells with large irregular hyperchromatic nuclei (Fig. 13) is very similar to those seen in the liver. The glomeruli are normal and there is no evidence of fibrosis.

*Adrenals*.—Within the first 24 hours there is congestion of the zona reticularis and some change to compact cells. The remainder of the cortex and medulla appear normal. Many animals by 48 hours show frank haemorrhage at the reticulo-medullary junction with compact cells extending through the zona fasciculata. Occasional mitoses may be seen in this zone. The extent and time of onset of the necrosis is very variable and can be seen 4–6 days after administration of the aflatoxin. The animals which survive 4 weeks have normal adrenals.

*Lungs*.—During the first few days after administration of the toxin the lungs are markedly congested with many petechial haemorrhages. At 1 month the lungs are normal.

*Heart.*—A few animals showed a patchy necrosis of the myocardium with a surrounding inflammatory reaction. This usually occurred during the first 4 days. At four weeks some of the animals show small areas of myocardial fibrosis.

*Spleen.*—Most animals showed a diminution of the white pulp progressing over 4–5 days. Occasional rats showed some necrosis of the red pulp immediately adjacent to the white pulp. This disappeared rapidly but resulted in a variable degree of fibrosis. After intraperitoneal injection many of the spleens showed a hyalinized perisplenitis.

*Alimentary tract.*—Animals dying within the first few days often had altered blood in the whole of the small gut and melaena stool in the colon. No obvious areas of ulceration could be seen.

*Pancreas.*—The acini and ducts were normal. In some animals the cells of the islets of Langerhans had pyknotic nuclei. By a month the pancreas was normal.

At the lowest dose level all the organs examined were normal. At the higher levels the haemorrhages seen in the lungs, kidneys and adrenals were more extensive.

### DISCUSSION

Aflatoxin is very insoluble in aqueous solution and is only partially soluble in methanol and ethanol. Although ethanol is a suitable vehicle for dosing day-old ducklings this is not the case with rats. The volume of ethanol required to dissolve up to 2 mg. of aflatoxin is lethal to rats. The solvent selected was dimethylformamide in which aflatoxin is readily soluble at least up to 20 mg. per ml. The toxicology of dimethylformamide has been studied by Heath (1962) but at the dose levels used, i.e. 0·1–0·2 ml. per rat no pathological changes would be expected.

Aflatoxin $B_1$ produces a periportal zone necrosis which develops slowly over a period of 48–72 hours. Only a few substances produce a periportal necrosis, for example, allyl alcohol and methylnitrosourethane (Schoental and Magee, 1962). Associated with the aflatoxin-induced lesion is a prominent biliary proliferation which is not seen with allyl alcohol. Remarkably few animals became jaundiced in spite of the well developed peripheral necrosis which seems to involve all the structures within the peripheral zone.

In contrast with most other hepatotoxic agents which produce central or periportal necrosis there is no rapid recovery. A month following a single dose of allyl alcohol which will cause a peripheral necrosis the liver is essentially normal. Following a single dose of carbon tetrachloride there is a rapid regeneration when many mitotic figures can be seen in the parenchymal cells (Cameron and Karunaratne, 1936). This rapid regeneration is not seen following aflatoxin. By four days a few mitoses can be seen in the parenchymal cells but are never abundant. The slow rate of regeneration is possibly related to the development of the enlarged parenchymal cell. These can be seen within about 7 days becoming more marked later.

After a month many of these large hyperchromatic parenchymal cells are present. Occasional abnormal mitoses can be identified in these cells. Similar cell types can be seen following administration of other hepatotoxic agents such as ethionine (Farber, 1956) and the pyrrolizidine alkaloids (Schoental and Magee, 1959). Similar cells also occur in rats fed aflatoxin in the diet. The nature of these cells is still uncertain. It was noticed during the feeding experiments that the large cells diminished in number after withdrawal of the toxic diet. The fate of these cells and the subsequent development of the liver lesion seen after one month is under investigation.

The continued biliary proliferation is most striking. Previously it has been reported that in the day-old duckling the extensive biliary proliferation regressed over a period of 10–14 days with rapid recovery of the parenchymal cells (Butler, 1964). It was suggested that aflatoxin might have a direct action upon the biliary epithelium and that the proliferation was a result of this and not secondary to the loss of parenchymal cells as suggested by Abercrombie and Harkness (1951). In the rat there is only a slow recovery of parenchymal cells. The prolonged liver insufficiency as a result of this may result in the continued biliary proliferation. After $CCl_4$ and allyl alcohol with rapid recovery no prolonged biliary proliferation is seen. As no evidence of biliary obstruction has been demonstrated this proliferation could be explained either by direct action on the biliary epithelium or by a prolonged liver insufficiency.

Although aflatoxin is a hepatocarcinogen many other organs are more or less severely affected in acute experiments. Many organs show small petechial haemorrhages in particular the adrenals and lungs. The kidney is interesting in that a tubular necrosis is seen within 48 hours which regenerates rapidly. By one month a few large atypical tubular cell nuclei can be seen. These large cells may be similar in behaviour to those seen in the liver. Tubular cells with atypical features have also been described in dichlorvinylcysteine poisoning (Parker and and Terracini, 1964).

### SUMMARY

The $LD_{50}$ of aflatoxin $B_1$ to male rats is estimated as 7 mg./kg. *per os* and 6 mg./kg. intraperitoneal and female rats 16 mg./kg. *per os*. The development of a periportal zone of necrosis over 3–4 days is described with a marked biliary proliferation. The lesion shows a slow recovery so that after 1 month the biliary proliferation persists as well as many large hyperchromatic parenchymal cells. The lesion in female rats is similar except that there is a greater accumulation of periportal fat before to the onset of necrosis. The pathological changes in the other organs are described.

It is a great pleasure to acknowledge the assistance of Dr. D. A. Van Dorp and Dr. J. de Iongh of Unilever Ltd., Vlaardingen, who generously supplied the purified aflatoxin.

I would like to thank Sir Roy Cameron and Dr. J. F. Smith for their advice and Mr. R. F. Coles and Miss M. Horn for their technical assistance. This work was supported by an M.R.C. grant to Dr. J. F. Smith and done during the tenure of the Graham Scholarship of the University of London.

These results were presented in part at the Symposium on Mycotoxins in Foodstuffs, Massachusetts Institute of Technology, March 1964.

### REFERENCES

ABERCROMBIE, M. AND HARKNESS, R. D.—(1951) *Proc. roy. Soc. B.*, **138**, 544.

ASAO, T., BÜCHI, G., ABDEL-KADER, M. M., CHANG, S. B., WICK, EMILY L. AND WOGAN, G. N.—(1963) *J. Amer. chem. Soc.*, **85**, 1706.

BUTLER, W. H.—(1964) *J. Path. Bact.*, **88**, 189.

BUTLER, W. H. AND BARNES, J. M.—(1963) *Brit. J. Cancer*, **17**, 699.
CAMERON, G. R. AND KARUNARATNE, W. A. E.—(1936) *J. Path. Bact.*, **42**, 1.
FARBER, E.—(1956) *Cancer Res.*, **16**, 142.
HEATH, D. F.—(1962) *Biochem. J.*, **85**, 72.
NESBITT, BRENDA F., O'KELLY, J., SARGEANT, K. AND SHERIDAN, Ann.—(1962) *Nature, Lond.*, **195**, 1062.
PARKER, V. H. AND TERRACINI, B.—(1964) *Food Cosmet. Toxicol.*, in press.
SCHOENTAL, REGINA AND MAGEE, P. N.—(1959) *J. Path. Bact.*, **78**, 471.
*Idem.*—(1962) *Brit. J. Cancer*, **16**, 92.
WEIL, C. S.—(1952) *Biometrics*, **8**, 3.